# Role of microRNA-4739 in enhancing cisplatin chemosensitivity by negative regulation of RHBDD2 in human cervical cancer cells

**DOI:** 10.1186/s11658-024-00532-6

**Published:** 2024-01-25

**Authors:** Yuling Li, Zhengtong Zhou, Jinfeng Qu, Peiling Gong, Yuyan Wei, Yaping Sun

**Affiliations:** 1 Department of Gynecology, Central Hospital Affiliated to Shandong First Medical University, No.105, Jiefang Road, Lixia District, Jinan, 250013 Shandong China; 2 Institute of Medical Genomics, Biomedical Sciences College & Shandong Medicinal Biotechnology Centre, Medical Science and Technology Innovation Center, Shandong First Medical University & Shandong Academy of Medical Sciences, Jinan, Shandong China; 3 Yiyuan Maternal and Child Health Hospital, Zibo, 256100 Shandong China

**Keywords:** Cervical cancer, miR-4739, Cisplatin, Drug resistance, RHBDD2

## Abstract

**Background:**

Cisplatin (DDP) is a widely used chemotherapy drug for advanced cervical cancer (CC), but resistance poses a significant challenge. While miR-4739 has been implicated in tumor development, its specific role in regulating DDP resistance in CC remains unclear.

**Methods:**

We analyzed the expression levels of miR-4739 and RHBDD2 in DDP-resistant and DDP-sensitive CC tissues using quantitative real-time polymerase chain reaction (PCR) and assessed their correlation through Spearman’s correlation analysis. DDP-resistant CC cell lines (HeLa/DDP and SiHa/DDP) were established by gradually increasing DDP concentrations, followed by transfection with miR-4739 mimics, si-RHBDD2, or a RHBDD2 overexpression vector. A series of functional assays, including CCK-8 assay, colony formation, flow cytometry, and transwell assay were performed. The interaction between miR-4739 and RHBDD2 was confirmed by luciferase reporter assay. We examined the protein levels of RHBDD2, P-gP, MRP1, cleaved caspase-3, and E-cadherin through western blot analysis. Moreover, we generated xenograft tumors by injecting stably transfected HeLa/DDP cells into mice to compare their tumorigenesis capacity.

**Results:**

We observed downregulation of miR-4739 and upregulation of RHBDD2 in DDP-resistant CC tissues and cell lines. MiR-4739 was shown to directly bind to RHBDD2 gene sequences to repress RHBDD2 expression in HeLa/DDP and SiHa/DDP cells. Our in vitro and in vivo experiments demonstrated that overexpressing miR-4739 overcame DDP resistance in CC cells by targeting RHBDD2. Furthermore, RHBDD2 overexpression reversed the effects of miR-4739 mimics on drug-resistance-related proteins (P-gP and MRP1) and the expression of cleaved caspase-3 and E-cadherin in HeLa/DDP cells.

**Conclusions:**

In summary, our study revealed that miR-4739 can reverse DDP resistance by modulating RHBDD2 in CC cells.

**Supplementary Information:**

The online version contains supplementary material available at 10.1186/s11658-024-00532-6.

## Background

Cervical cancer (CC) is widely recognized as a prominent malignancy in females, posing a significant public health challenge on a global scale [1]. It holds the fourth position in terms of incidence and mortality among gynecological cancers worldwide, drawing considerable attention and concern, as reported in published studies [2]. Concurrent chemoradiotherapy based on cisplatin (DDP) is the established standard treatment for patients with CC [3]. Unfortunately, the effectiveness of this approach is frequently compromised by the development of inherent or acquired resistance to DDP, presenting a significant hurdle in achieving optimal treatment outcomes [4, 5]. Hence, it is crucial to explore the underlying mechanisms of DDP resistance in CC and enhance DDP sensitivity to improve the treatment efficacy for patients with CC.

Resistance to DDP involves diverse mechanisms, including reduced intracellular drug accumulation, suppression of apoptosis, and promotion of epithelial–mesenchymal transition (EMT) [6, 7]. Intriguingly, recent evidence suggests that DDP resistance can stem from epigenetic alterations at the molecular and cellular levels, notably through aberrant expression of microRNAs (miRNAs/miRs) [8]. MiRNAs are small RNA molecules, less than 25 nucleotides in length, that can attach to the 3′-untranslated region (3′-UTR) of specific mRNA targets. This binding leads to the degradation and suppression of the targeted mRNAs [9]. Consequently, miRNAs can act as oncogenes or tumor suppressors in the development of tumors. In the field of tumor malignancy, researchers have discovered numerous miRNA molecules and their corresponding target genes, which play significant roles in regulating chemoresistance [10–12]. Recent research has indicated that miR-4739 is downregulated in gastric cancer [13], prostate cancer [14], and lung adenocarcinoma [15], while it is upregulated in hepatocellular carcinoma [16]. In terms of functionality, increased expression of miR-4739 has been observed to inhibit the growth of esophageal squamous cell carcinoma (ESCC) cells by suppressing the expression of homeobox C10 [17]. Nevertheless, there is a lack of information regarding the function and molecular mechanism of miR-4739 in relation to chemoresistance in CC cells.

Rhomboid pseudoproteases are noncatalytic members of the rhomboid superfamily that regulate the trafficking, turnover, and activity of target proteins [18]. Among them, rhomboid domain containing 2 (RHBDD2) is an intramembrane pseudoprotease member of the rhomboid family that shows elevated expression levels during mammary gland development and in advanced tumor stages [19, 20]. In a recent study by Zhu et al. [21], RHBDD2 was identified as one of the newly discovered candidates in individuals affected by familial nonmedullary thyroid cancer. A notable study by Abba et al. [20] revealed that targeted inhibition of RHBDD2 expression using siRNA resulted in decreased proliferation of MCF-7 breast cancer cells compared with the control group. Intriguingly, Palma et al. [22] confirmed that elevated expression of RHBDD2 in locally advanced rectal cancer tumors following neoadjuvant radiochemotherapy (NRC) treatment is linked to the development of both local and distant metastases. Our bioinformatics analysis indicated that RHBDD2 is a potential target gene of miR-4739. This discovery led us to formulate the hypothesis that miR-4739 plays a role in regulating DDP resistance in CC by targeting RHBDD2. To investigate this hypothesis, the present study was designed with the aim of exploring the relationship between miR-4739 and RHBDD2 in the context of DDP resistance.

To begin our study, we established two DDP-resistant CC cell lines (HeLa/DDP and SiHa/DDP) and assessed the expression levels of miR-4739 and RHBDD2 in clinical tissues and cells. Subsequently, we conducted a series of gain-of-function, loss-of-function, and rescue experiments to delve into the underlying mechanism by which the miR-4739/RHBDD2 axis contributes to chemoresistance. Our study endeavors to shed light on the significant role played by the miR-4739/RHBDD2 axis in the development of chemoresistance in CC, providing valuable insights in the process.

## Materials and methods

### Tissue samples

This study involved a total of 70 patients with CC who underwent DDP-based chemotherapy at the Department of Gynecology in the Central Hospital Affiliated to Shandong First Medical University, located in Shandong, China. The patient group consisted of an equal number of individuals with DDP-sensitive tissues (tumor remission after six cycles of DDP chemotherapy; *n* = 35) and DDP-resistant tissues (tumor stabilization or progression after six cycles of DDP chemotherapy; *n* = 35). No patients underwent radiotherapy or immunotherapy before surgery. Before participating in the study, each patient provided written informed consent. The collected tissue samples were stored at −80 °C until further analysis. Approval for this research was obtained from the Research Ethics Committee of the Central Hospital Affiliated to Shandong First Medical University.

### Cell lines and culture

We procured two CC cell lines, HeLa and SiHa, from ATCC (Manassas, VA). These cell lines were cultivated in DMEM/F12 medium (Sigma-Aldrich, St. Louis, MO) supplemented with 10% fetal bovine serum (FBS) obtained from Gibco (Grand Island, NY). The cells were incubated at 37 °C in a humidified incubator with 5% CO_2_.

### Generation of DDP-resistant cell lines

DDP, with a minimum purity of > 98%, was obtained from Sigma-Aldrich (St. Louis, MO) and reconstituted in distilled water to achieve suitable concentrations. HeLa and SiHa cells were subjected to escalating concentrations of DDP to establish DDP-resistant cell lines. The parental HeLa and SiHa cells in the exponential growth phase were initially cultured in a medium containing 0.1 μg/ml of DDP. After 14 days, the DDP concentration was incrementally increased to 0.3 µg/ml for an additional 2-week incubation period. Subsequently, the DDP concentration was gradually raised to 0.6 µg/ml and 1.2 µg/ml until the cells exhibited stable growth and were passaged repeatedly. Approximately 10 months later, DDP-resistant cell lines (HeLa/DDP and SiHa/DDP) were successfully generated and maintained in a medium containing DDP to preserve their resistance characteristics.

### Cell transfection and treatment

HeLa/DDP and SiHa/DDP cells were transfected with miR-4739 mimics and specific small interfering RNA targeting RHBDD2 (si-RHBDD2), as well as negative control sequences (NC mimics and si-NC). These molecules were synthesized by GenePharma Company (Shanghai, China). The pcDNA3.1–RHBDD2 expression vector, which carries the human RHBDD2-coding DNA sequence, was cloned into a pcDNA3.1 vector obtained from Thermo Fisher Scientific, Inc. In the rescue experiments, HeLa/DDP cells were cotransfected with miR-4739 mimics and the pcDNA3.1–RHBDD2 vector. For the in vitro functional analysis, HeLa/DDP or SiHa/DDP cells from different transfection groups underwent with or without treatment with 2 μg/ml DDP for 24 h. Lipofectamine 3000 transfection reagent from Invitrogen was employed for all transfections for 48 h, adhering strictly to the manufacturer’s instructions.

### CCK-8 assay

DDP-resistant HeLa/DDP and SiHa/DDP cells were seeded into 96-well plates and incubated for 24 h. Subsequently, the cells were subjected to DDP treatment at concentrations of 1, 2, 3, 4, or 5 μg/ml, along with any required transfections. After an additional 48 h of incubation, 10 μl of Cell Counting Kit-8 (CCK-8) agent from Doujindo Laboratories (Tokyo, Japan) was added to each well and incubated for 2 h at 37 °C. The absorbance was measured at 450 nm using a BioTek microplate reader (Winooski, VT). The inhibition rate was calculated using the formula: Inhibition rate = (1-A450 value of the treated group)/A450 value of the control group × 100%. The half-maximal inhibitory concentration (IC50) was determined using GraphPad software, based on the CCK-8 results, to ascertain the drug concentration necessary for a 50% inhibition of cell growth.

### Colony formation assay

HeLa/DDP and SiHa/DDP cells from various groups were seeded in 6-well plates at a density of 500 cells per well. The cells were cultured for approximately 2 weeks at 37 °C in a 5% CO_2_ incubator. After washing with phosphate-buffered saline (PBS), the cells were fixed in 4% paraformaldehyde for 15 min and then stained with 0.1% crystal violet for 30 min. The visible colonies were captured and counted under a microscope (Olympus, Tokyo, Japan).

### Cell apoptosis assessment

HeLa/DDP or SiHa/DDP cells from different experimental groups were seeded in 60-mm dishes at a density of 5 × 10^5^ cells per dish and cultured for 48 h. The cells were then harvested by trypsinization and washed twice with ice-cold PBS. Subsequently, the cells were resuspended in binding buffer at a concentration of 1 × 10^6^ cells per ml. The cell suspension was stained with 5 μl of Annexin V-FITC (51-65874X; BD, Franklin Lakes, NJ) and 5 μl of propidium iodide (PI; 51-66211E; BD) in the dark at 25 °C. Cell apoptosis assessment was conducted using flow cytometry (FACSAria Cell Sorter, BD Biosciences, USA) within 1 h.

### Cell migration assay

Cell migration experiments were conducted using a 24-well transwell chamber assay with a pore size of 8 µm (BD Biosciences). Briefly, HeLa/DDP or SiHa/DDP cells from various experimental groups were trypsinized to obtain single-cell suspensions. The upper chamber of the transwell was seeded with 2 × 10^4^ cells in 100 µl of serum-free medium, while the lower chamber was filled with 500 µl of complete medium containing 20% FBS as a chemoattractant. After 24 h, the migrated cells remaining on the lower chamber were fixed with 4% paraformaldehyde, stained with 0.1% crystal violet, and counted using a light microscope.

### Prediction of miR-4739 targets

Using TargetScan software (release 7.2; http://www.targetscan.org/vert_72/), we conducted a search for potential targets of miR-4739. Among the results, RHBDD2 was identified as a candidate target.

### Luciferase reporter assay

The luciferase reporter psiCHECK-2 vector was utilized to synthesize the wild-type 3′-untranslated region (3′-UTR) sequences of RHBDD2 cDNA [wildtype (WT)-RHBDD2]. Additionally, a mutant RHBDD2 3′-UTR vector (MUT-RHBDD2) was created with a specific mutation in the predicted RHBDD2-binding sequence. HeLa/DDP or SiHa/DDP cells were seeded in 24-well plates and cotransfected with 500 ng of the respective vectors along with 100 nM of either miR-4739 mimics or NC mimics using Lipofectamine 3000 reagent. Following a 48-h incubation, the Luciferase-Reporter Assay System kit from Promega (Madison, WI) was employed to measure luciferase activity, using Renilla luciferase activity as the internal reference.

### Western blot analysis

To extract total protein, cell lysis was performed using a RIPA buffer from Beyotime Institute of Biotechnology, and the concentration of the extracted protein was determined using a BCA assay kit, also from Beyotime Institute of Biotechnology. Protein extracts (30 μg) were loaded onto a sodium dodecyl-sulfate (SDS)–polyacrylamide gel for separation and subsequently transferred to polyvinylidene fluoride (PVDF) membranes. Following membrane blocking with 5% nonfat milk at room temperature for 1 h, primary antibodies against RHBDD2, P-gP, MRP1, cleaved caspase-3, E-cadherin, and GAPDH (from Abcam, Cambridge, MA) were added to the membrane and incubated overnight at 4 °C. The membranes were then incubated with corresponding secondary antibodies at room temperature. Protein detection was performed using an enhanced chemiluminescence (ECL) detection kit from Amersham Pharmacia Biotech (UK).

### Xenograft mice assay in vivo

A total of 15 BALB/c female nude mice (4 weeks old, weighing 15–20 g) were obtained from Shanghai Laboratory Animal Center. The mice were housed in a comfortable environment with controlled conditions, including a temperature range of 21–24 °C, relative humidity of 50–60%, and a 12-h light/dark cycle. They were provided with unrestricted access to food and water. HeLa/DDP cells were prepared with overexpressed miR-4739, NC mimics, and overexpressed miR-4739 and RHBDD2. Cells from each group (1 × 10^7^) were mixed with Matrigel (BD Biosciences, USA) and injected into the right flanks of the mice to induce xenograft tumors. Consequently, all mice were divided into three groups, with five mice in each group. When the tumor volumes reached approximately 100 mm^3^, the mice were intraperitoneally injected with DDP (20 mg/kg) on a daily basis for 28 days. Tumor volume was monitored continuously for 42 days by measuring the length and width, with tumor volume calculated using the formula (length × width^2^)/2. After 42 days of observation, all nude mice were euthanized, and the tumor tissues were isolated, photographed, and subjected to immunohistochemistry and quantitative real-time PCR analysis.

### Immunohistochemistry

The excised tumor tissues were fixed in 4% paraformaldehyde and subsequently embedded in paraffin. The paraffin-embedded tissues were then sectioned to a thickness of 4 µm. These sections were deparaffinized and rehydrated using a series of graded alcohols, followed by two washes in PBS for 10 min each. Next, the sections were incubated overnight with a rabbit polyclonal primary antibody specific to RHBDD2. Afterward, the sections were treated with a horseradish peroxidase-conjugated secondary antibody for 1 h at 37 °C. To visualize the antibody binding, the sections were stained with a 3,3′-diaminobenzidine (DAB) working solution for 3 min, washed in water for 10 min, and counterstained with hematoxylin. Subsequently, the sections were rinsed again in water for 10 min, followed by dehydration and clearing of the cross-sections. Finally, the sections were observed and photographed using the BX43 microscope system (Olympus, Japan).

### Quantitative real time PCR

Total RNA was extracted from tissues or cultured cells using TRIzol reagent (Invitrogen, Carlsbad, USA). The Mir-X™ miRNA First Strand Synthesis Kit and PrimeScript RT Reagent Kit (Invitrogen) were utilized to perform reverse transcription of miRNA and mRNA into cDNA, respectively. For miR-4739 detection, quantitative real-time PCR was conducted using the SYBR PrimeScriptTM miRNA RT-PCR Kit, while the SYBR Premix Ex Taq II (Takara, Dalian, China) was used for RHBDD2 mRNA detection. The thermocycling conditions consisted of an initial denaturation step at 95 °C for 10 min, followed by 40 cycles of denaturation at 92 °C for 30 s and anneal/extend at 60 °C for 35 s. The primer sequences used were as follows: miR-4739 forward: 5′-AAGGGAGGAGGAGCGGAG-3′ and reverse: 5′-GAACATGTCTGCGTATCTC-3’; U6 forward: 5′-CTCGCTTCGGCAGCACATATACT-3 and reverse: 5′-ACGCTTCACGAATTTGCGTGTC-3′; RHBDD2 forward: forward: 5′- GAGGCCCTTCGCAACTGG-3′ and reverse: 5′-GCAGGGAGATGGGATTCTCG-3’; GAPDH forward: 5′-GGTGAAGGTCGGAGTCAACG-3 and reverse: 5′-GCATCGCCCCACTTGATTTT-3′. The relative expression levels of miR-4739 and RHBDD2 were determined using the comparative Ct method (2^−ΔΔCt^). The expression levels were normalized to U6 for miR-4739 and GAPDH for RHBDD2, respectively.

### Statistical analysis

All experiments were conducted with a minimum of three independent repetitions, and each assay included triplicate samples. The data are expressed as the mean ± standard deviation (SD). Statistical analysis was performed using GraphPad Prism software version 7.0 (GraphPad Software, Inc.). Differences between groups were assessed using Student’s *t*-test. For comparisons among more than two groups, one-way analysis of variance (ANOVA) with Tukey’s multiple comparison post hoc test was employed. Spearman’s correlation analysis was used to evaluate the correlation between RHBDD2 mRNA and miR-4739. A *p*-value less than 0.05 was considered statistically significant.

## Results

### The dysregulation of miR-4739 and RHBDD2 were associated with the DDP resistance of CC

**Fig. 1:**
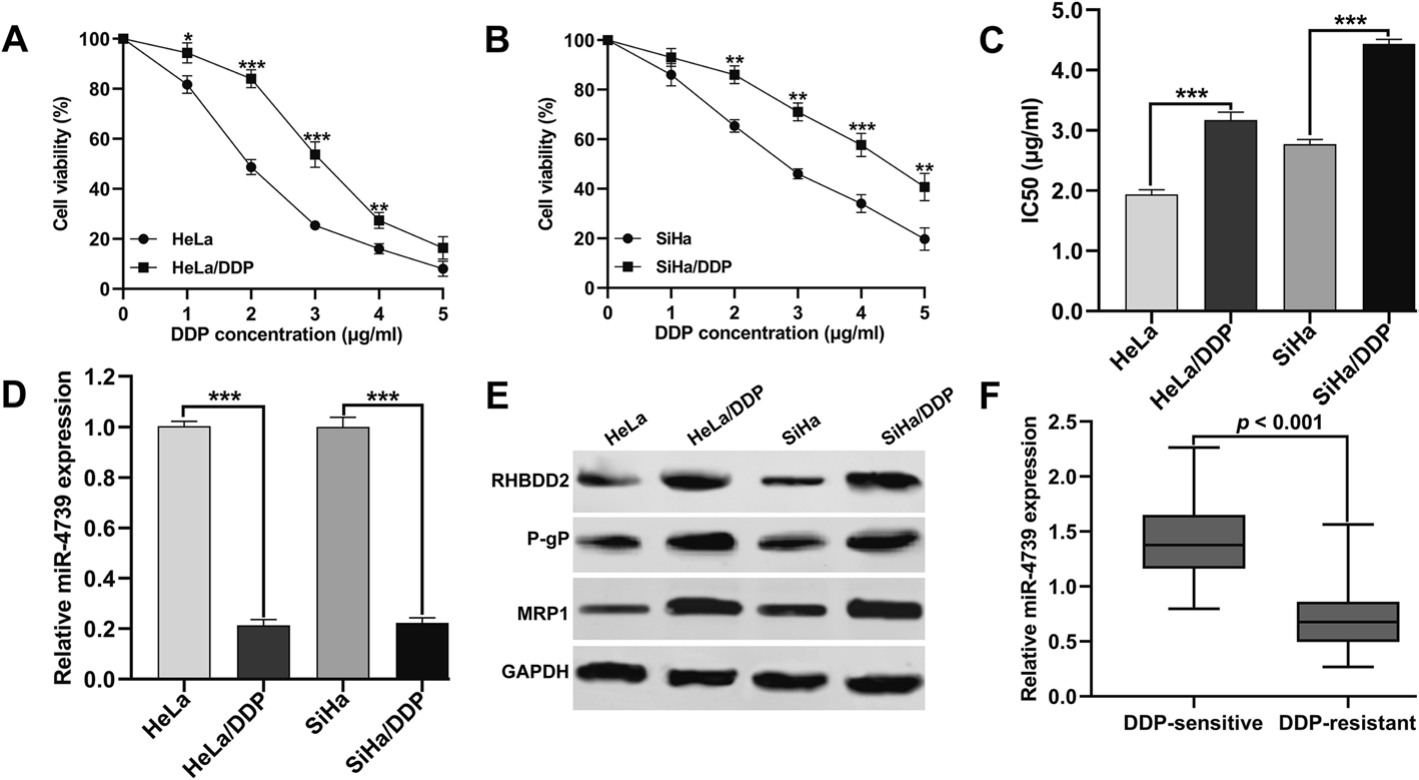
The dysregulation of miR-4739 and RHBDD2 were associated with the DDP resistance of CC. **A**, **B** Parental CC cell lines (HeLa and SiHa) and their corresponding DDP-resistant cell lines (HeLa/DDP and SiHa/DDP) were exposed to 1, 2, 3, 4, 5 μg/ml DDP and the cell viability was determined using CCK-8 assay. **C** IC50 values of HeLa, HeLa/DDP, SiHa and SiHa/DDP cells were calculated and shown. **D** The miR-4739 expression levels in HeLa, HeLa/DDP, SiHa and SiHa/DDP cells were determined using quantitative real time PCR. **E** The protein levels of RHBDD2, P-gP, and MRP1 were detected via western blot analysis in HeLa, HeLa/DDP, SiHa, and SiHa/DDP cells. **F** MiR-4739 expression in DDP-sensitive and DDP-resistant CC tissues both from patients with CC (*n* = 35) was determined using quantitative real-time PCR. *p < 0.05, **p < 0.01, ***p < 0.001, compared with HeLa or SiHa via Student’s *t*-test

To investigate the role of miR-4739 and RHBDD2 in DDP resistance in CC, we initially analyzed DDP-resistant CC cell lines derived from HeLa and SiHa cells. The DDP resistance of these cell lines was confirmed by exposing the parental CC cell lines (HeLa and SiHa) and their corresponding DDP-resistant cell lines (HeLa/DDP and SiHa/DDP) to increasing concentrations of DDP (1, 2, 3, 4, or 5 μg/ml) and evaluating the IC50 values using cell viability assays. The results from the CCK-8 assay demonstrated that the cell viability of HeLa/DDP and SiHa/DDP cells was significantly higher than that of the corresponding parental HeLa (Fig. 1A) and SiHa cells (Fig. 1B). Consequently, the IC50 values for HeLa/DDP and SiHa/DDP were significantly higher compared with the original HeLa and SiHa cells (Fig. 1C). Moreover, a notable reduction in the expression level of miR-4739 was observed in the DDP-resistant cells we established, as compared with their corresponding parental cells (Fig. 1D). Furthermore, western blot analysis revealed the upregulation of RHBDD2, P-gP, and MRP1 in HeLa/DDP and SiHa/DDP cells compared with HeLa and SiHa cells, respectively (Fig. 1E). Additionally, we analyzed the expression levels of miR-4739 in DDP-resistant tissues from CC patients. Consistently, miR-4739 expression was decreased in DDP-resistant CC tissues in comparison with those in DDP-sensitive samples (Fig. 1F).

### Overexpression of miR-4739 enhanced the chemosensitivity of CC cells to DDP in vitro

**Fig. 2:**
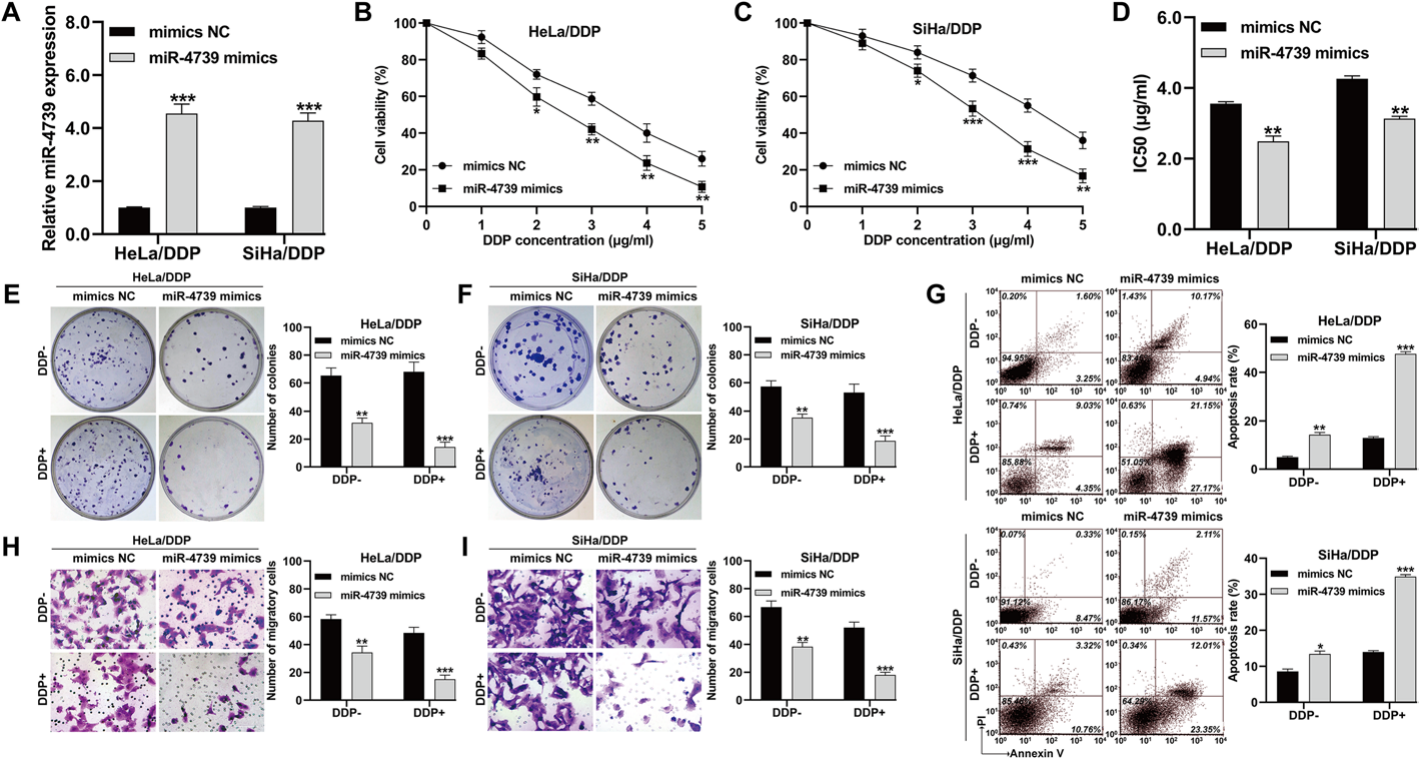
MiR-4739 enhanced the chemosensitivity of CC cells to DDP in vitro. HeLa/DDP and SiHa/DDP cells were transfected with miR-4739 mimics or NC mimics for 48 h and then subjected to treatment with or without 2 μg/ml DDP for 24 h. **A** The miR-4739 expression levels were determined in the above HeLa/DDP and SiHa/DDP cells. **B**, **C** The cell viability of the above HeLa/DDP and SiHa/DDP cells was determined using CCK-8 assay. **D** IC50 values of the above HeLa/DDP and SiHa/DDP cells were calculated and shown. **E**, **F** Colony formation assay. **G** Cell apoptosis rate was determined by flow cytometry assay. **H**, **I** Transwell assay was applied to analyze cell migration ability of the above HeLa/DDP and SiHa/DDP cells. *p < 0.05, **p < 0.01, ***p < 0.001, compared with NC mimics via Student’s *t*-test

To investigate the impact of miR-4739 modulation on the chemosensitivity of DDP-resistant CC cells, we conducted gain-of-function assays in DDP-resistant CC cells. Initially, HeLa/DDP and SiHa/DDP cells were transfected with miR-4739 mimics to establish DDP-resistant CC cell lines with stable high expression of miR-4739, as confirmed by quantitative real-time PCR analysis (Fig. 2A). Subsequently, we evaluated the effect of miR-4739 overexpression on cell viability using the CCK-8 assay. The results revealed that the upregulation of miR-4739 significantly suppressed the cell viability of HeLa/DDP (Fig. 2B) and SiHa/DDP (Fig. 2C) cells under treatment with increasing DDP concentration. Moreover, the IC50 value was notably reduced in HeLa/DDP and SiHa/DDP cells with miR-4739 overexpression compared with the negative control (Fig. 2D). Furthermore, subsequent experiments demonstrated that miR-4739 overexpression inhibited colony formation (Fig. 2E, F), increased the apoptosis rate (Fig. 2G), and impaired the migration ability (Fig. 2H, I) of HeLa/DDP and SiHa/DDP cells, with more pronounced effects observed after DDP treatment (Additional file 1: Fig. S1A–C).

### RHBDD2 was a direct target of miR-4739

**Fig. 3:**
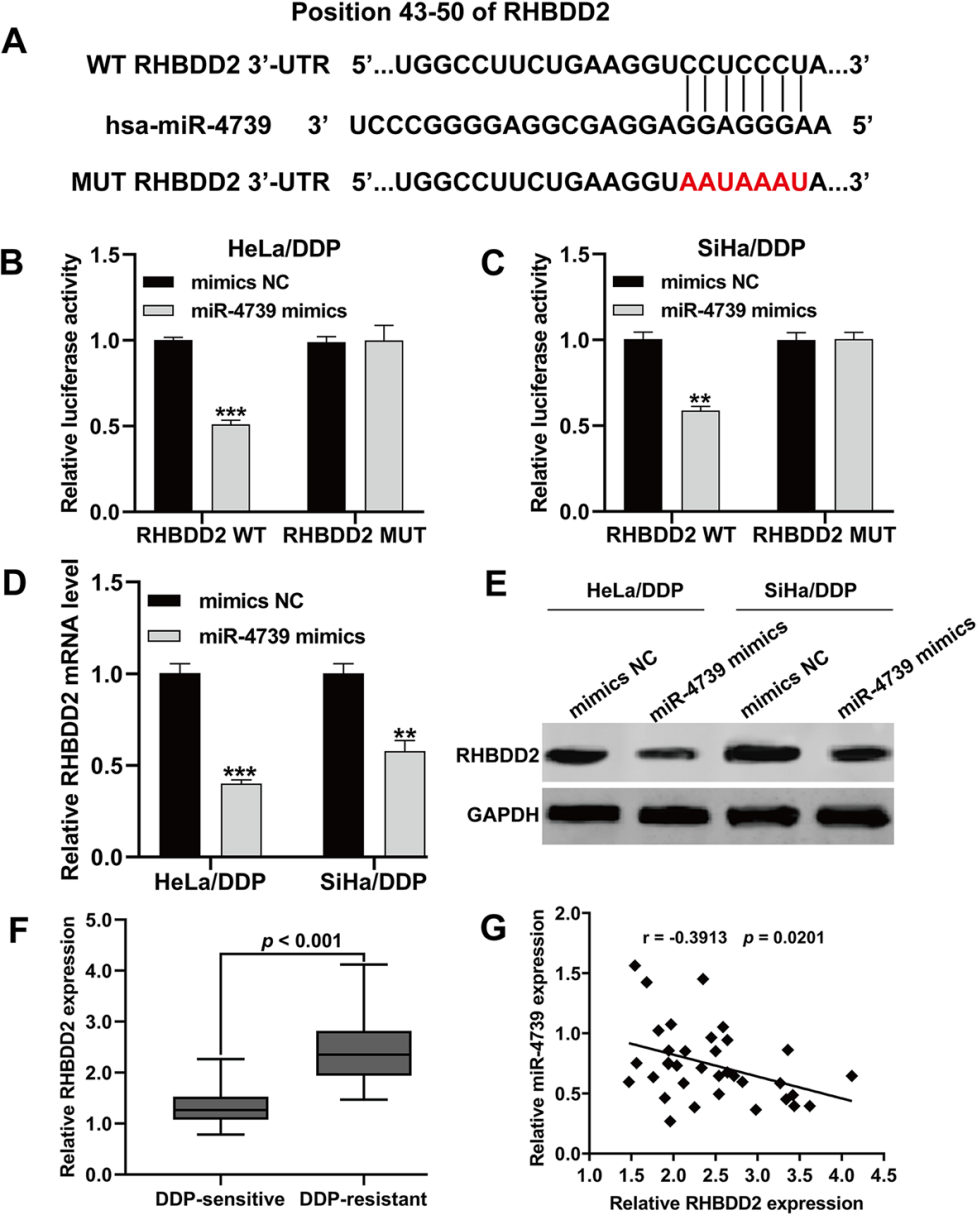
RHBDD2 was a direct target of miR-4739. **A** Predicted binding site between miR-4739 and RHBDD2 3′-UTR. **B**, **C** Luciferase activity for HeLa/DDP and SiHa/DDP cells cotransfected with WT-RHBDD2 or MUT-RHBDD2 and miR-4739 mimics or NC mimics. **D** Relative expression of RHBDD2 mRNA in HeLa/DDP and SiHa/DDP cells transfected with miR-4739 mimics or NC mimics were determined by quantitative real time PCR. **p < 0.01, ***p < 0.001, compared with NC mimics via Student’s *t*-test. **E** RHBDD2 protein levels in the transfected cells were evaluated by western blot analysis and GAPDH was used as the control. **F** The expression of RHBDD2 was determined via quantitative real-time PCR in DDP-resistant (*n* = 35) and DDP-sensitive tissues (*n* = 35) both from CC patients. **G** Spearman rank correlation analysis showed an inverse correlation between the expression levels of RHBDD2 and miR-4739 in DDP-resistant tissues from patients with

To investigate the underlying mechanisms of miR-4739 in regulating DDP-resistant CC cells, we utilized TargetScan software to identify potential targets of miR-4739, leading us to select the *RHBDD2* gene as a potential candidate (Fig. 3A). Subsequently, we performed luciferase assays to examine whether miR-4739 directly suppressed the expression of *RHBDD2* in DDP-resistant cells. The luciferase activity of the reporter vector containing the wild-type RHBDD2 3′-UTR was significantly inhibited by miR-4739 mimics, while the luciferase activity of the reporter construct containing the mutant RHBDD2 3′-UTR remained unaffected in both HeLa/DDP (Fig. 3B) and SiHa/DDP (Fig. 3C) cells. Additionally, transfection with miR-4739 mimics resulted in a reduction in the mRNA (Fig. 3D) and protein (Fig. 3E) levels of RHBDD2 in HeLa/DDP and SiHa/DDP cells. Thus, miR-4739 was shown to directly bind to RHBDD2 gene sequences to repress RHBDD2 expression in HeLa/DDP and SiHa/DDP cells. Furthermore, the mRNA expression of RHBDD2 was significantly upregulated in DDP-resistant tissues from patients with CC compared with DDP-sensitive tissues from patients with CC (Fig. 3F). Notably, there was an inverse correlation observed between the expression of miR-4739 and RHBDD2 in DDP-resistant tissues from patients with CC (Fig. 3G).

### Knockdown of RHBDD2 promoted the chemosensitivity of CC cells to DDP in vitro

**Fig. 4:**
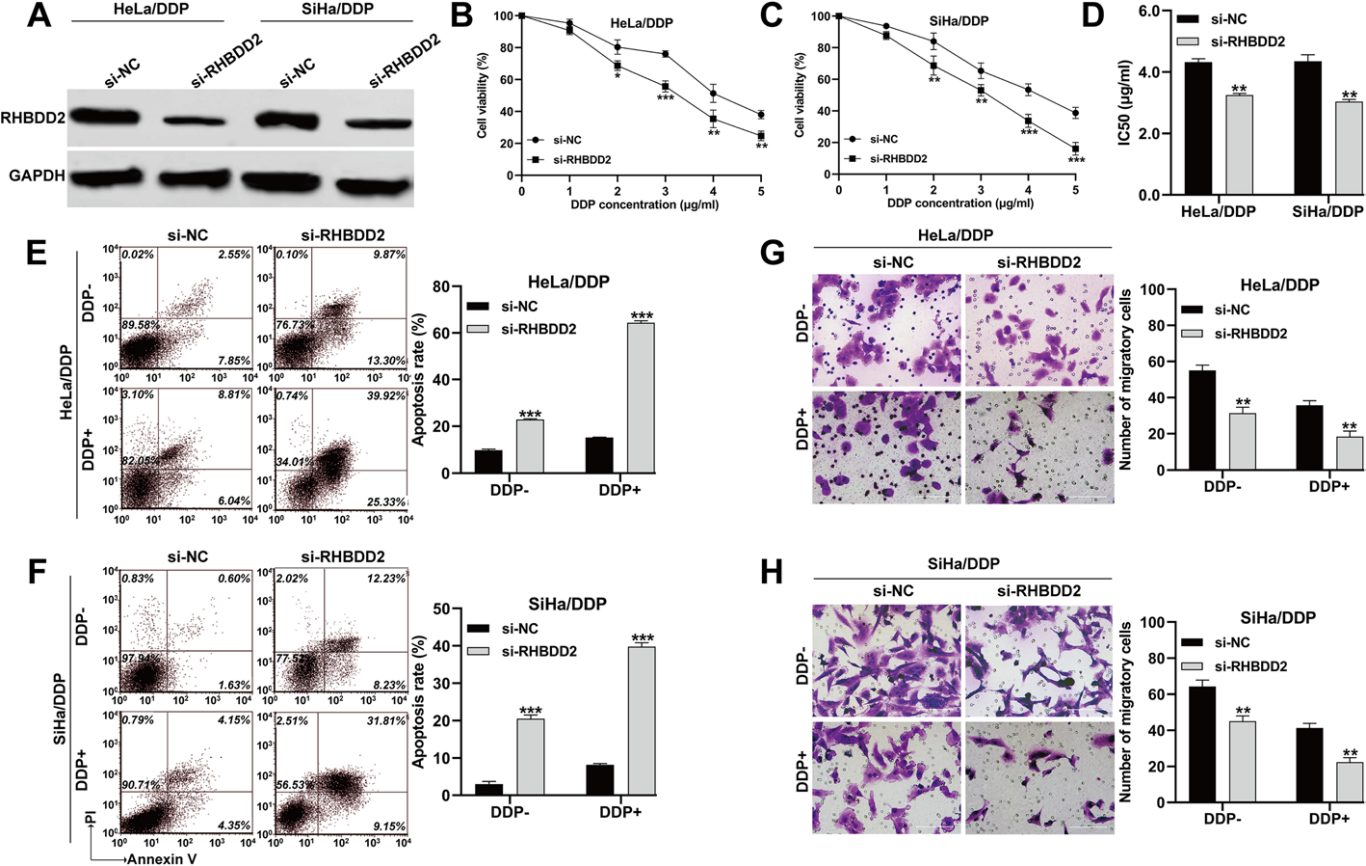
Knockdown of RHBDD2 promoted the chemosensitivity of CC cells to DDP in vitro. HeLa/DDP and SiHa/DDP cells were transfected with si-RHBDD2 or si-NC for 48 h, and then subjected to treatment with or without 2 μg/ml DDP for 24 h. **A** The expression of RHBDD2 mRNA levels were determined in the above HeLa/DDP and SiHa/DDP cells. **B**, **C** The cell viability of the above HeLa/DDP and SiHa/DDP cells was determined using CCK-8 assay. **D** IC50 values of the above HeLa/DDP and SiHa/DDP cells were calculated and shown. **E**, **F** Cell apoptosis rate was determined by flow cytometry assay. **G**, **H** Transwell assay was applied to analyze cell migration ability of the above HeLa/DDP and SiHa/DDP cells. *p < 0.05, **p < 0.01, ***p < 0.001, compared with si-NC via Student’s *t*-test

To investigate the impact of RHBDD2 on DDP-resistant CC, we performed stable knockdown of RHBDD2 expression in HeLa/DDP and SiHa/DDP cell lines using si-RHBDD2. Western blot analysis confirmed a significant decrease in RHBDD2 protein expression in HeLa/DDP and SiHa/DDP cells following si-RHBDD2 transfection (Fig. 4A). Consistent with the effects of miR-4739 overexpression, knockdown of RHBDD2 led to a significant reduction in cell viability in HeLa/DDP (Fig. 4B) and SiHa/DDP (Fig. 4C) cells upon treatment with increasing concentrations of DDP, resulting in decreased IC50 values for both cell lines (Fig. 4D). Furthermore, RHBDD2 knockdown promoted apoptosis (Fig. 4E, F) and suppressed migration ability (Fig. 4G, H) in HeLa/DDP and SiHa/DDP cells, particularly following DDP treatment (Additional file 1: Figure S1D, E).

### Overexpression of RHBDD2 reversed the regulatory of miR-4739 on DDP resistance in CC cells

**Fig. 5:**
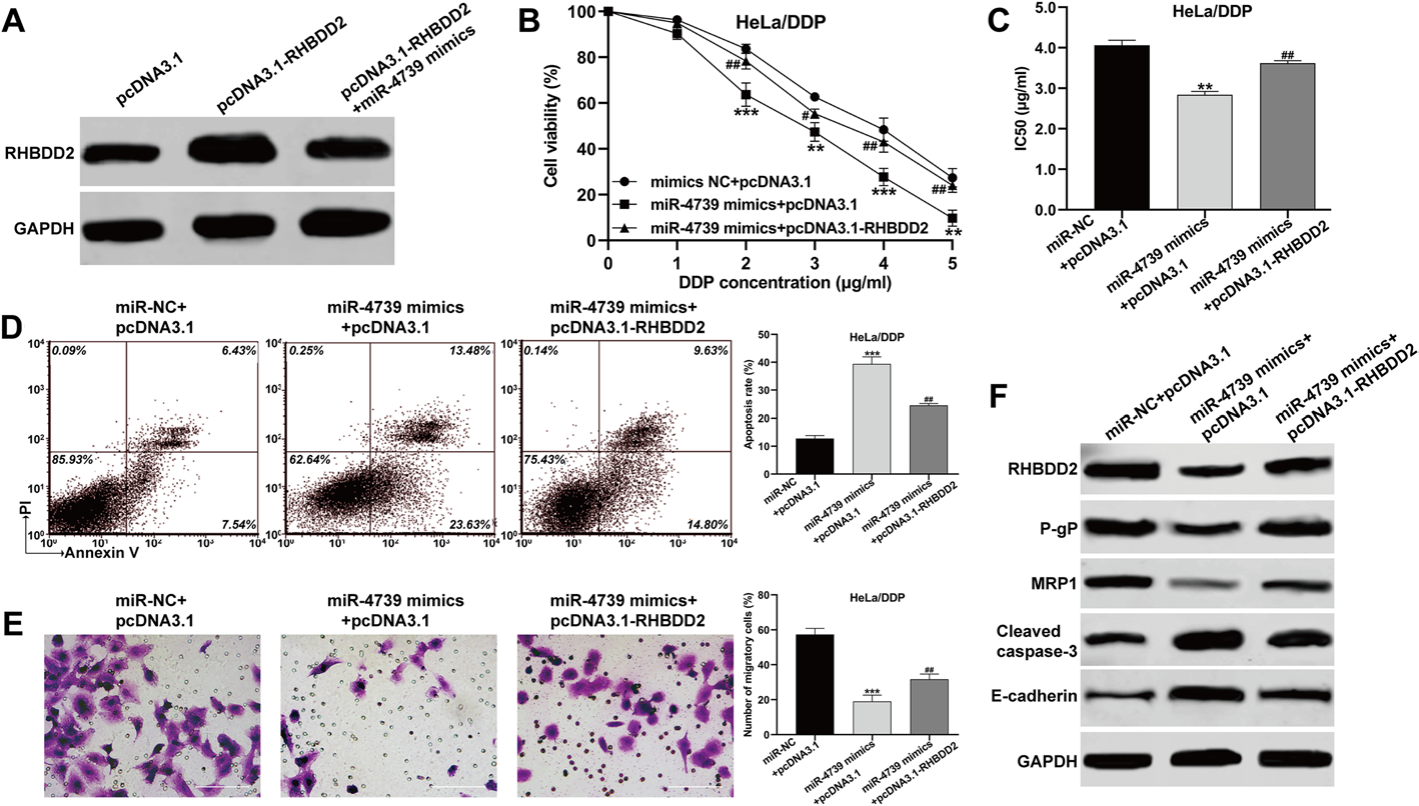
Overexpression of RHBDD2 reversed the regulatory of miR-4739 on DDP resistance in CC cells. HeLa/DDP cells were transfected with pcDNA3.1, pcDNA3.1–RHBDD2, or pcDNA3.1–RHBDD2 along with miR-4739 mimics. **A** The protein expression of RHBDD2 was detected by western blot analysis. **B** The cell viability of the transfected HeLa/DDP cells was determined using CCK-8 assay. **C** IC50 values of the transfected HeLa/DDP cells were calculated and shown. **D** Cell apoptosis rate was determined by flow cytometry assay. **E** Transwell assay was applied to analyze cell migration ability of the transfected HeLa/DDP cells. **F** The protein levels of RHBDD2, P-gP, MRP1, cleaved caspase-3, and E-cadherin were determined by western blot analysis. **p < 0.01, ***p < 0.001, compared with NC mimics + pcDNA3.1; #*p* < 0.05, ##*p* < 0.01, compared with miR-4739 mimics + pcDNA3.1

To confirm the involvement of RHBDD2 as a downstream regulator in miR-4739-mediated regulation of DDP-resistance in CC cells, experiments were performed. HeLa/DDP cells were transfected with pcDNA3.1, pcDNA3.1–RHBDD2, or pcDNA3.1–RHBDD2 along with miR-4739 mimics. Western blot analysis showed that the overexpression of RHBDD2 using pcDNA3.1–RHBDD2 was diminished upon miR-4739 overexpression (Fig. 5A). The CCK-8 assay results demonstrated that miR-4739 mimics reduced cell viability (Fig. 5B) and lowered the IC50 values (Fig. 5C) of HeLa/DDP cells, indicating increased sensitivity to DDP. However, the effects of miR-4739 mimics were reversed when RHBDD2 was overexpressed. Furthermore, miR-4739 mimics elevated the apoptosis rate of HeLa/DDP cells, whereas overexpression of RHBDD2 counteracted the effects induced by miR-4739 mimics (Fig. 5D). Similarly, the reduced cell migration caused by miR-4739 mimics was significantly restored after RHBDD2 overexpression in HeLa/DDP cells (Fig. 5E). Detailed analysis using western blot showed that RHBDD2 overexpression counteracted the inhibitory impact of miR-4739 mimics on both RHBDD2 itself and the expression of drug-resistance-related proteins (P-gP and MRP1). Moreover, the increase in cleaved caspase-3 and E-cadherin levels induced by miR-4739 mimics was also counteracted when the amount of RHBDD2 was increased in HeLa/DDP cells (Fig. 5F). Overall, these results indicate that miR-4739 enhances the sensitivity of CC cells to DDP by targeting RHBDD2.

### MiR-4739 overexpression overcome DDP resistance in CC cells by targeting RHBDD2 in vivo

**Fig. 6:**
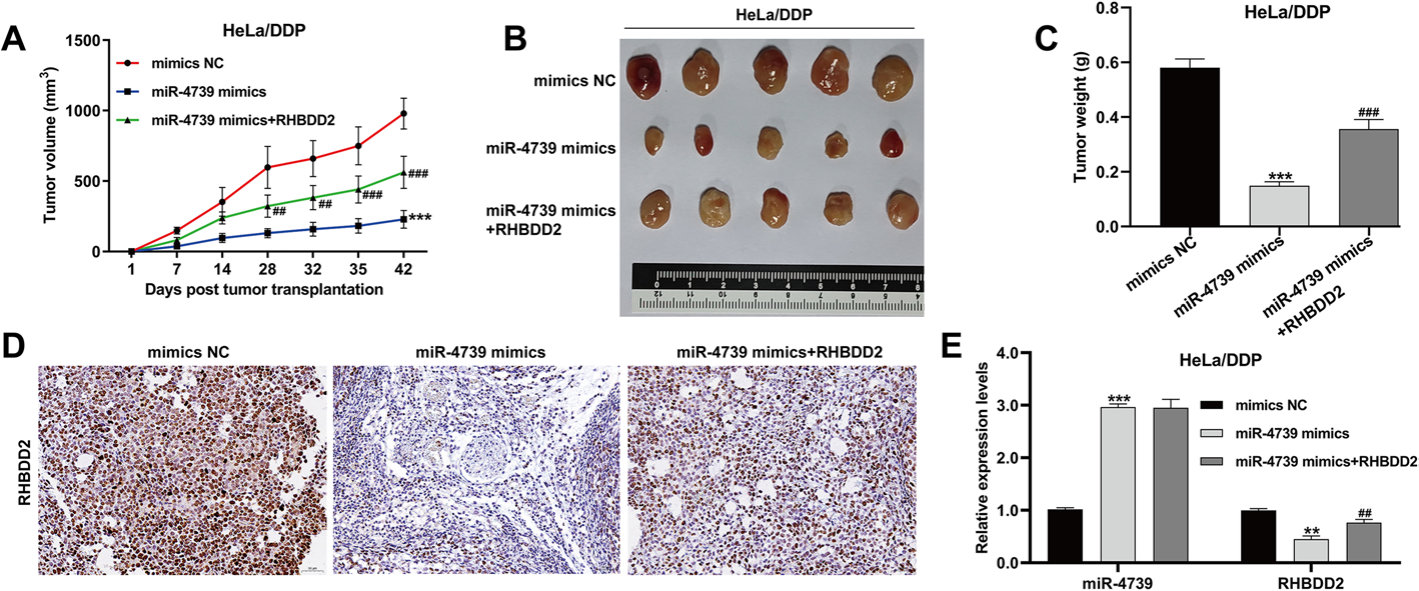
MiR-4739 overexpression overcome DDP resistance in CC cells by targeting RHBDD2 in vivo. BALB/c nude mice were injected with HeLa/DDP cells (1 × 10^7^) transfected with miR-4739 mimics, NC mimics, and miR-4739 mimics + RHBDD2, respectively (*n* = 5 per group). After 2 weeks (day 14), the mice were intraperitoneally administered with DDP (20 mg/kg) every week for 4 weeks. **A** Tumor volume was monitored continuously for 42 days. On day 42, the xenografts were resected (**B**) and weighed (**C**). **D** The representative images of RHBDD2 expression by immunohistochemical stain were shown in xenograft tumor tissues. Scale bar, 50 µm. **E** The expression of miR-4739 and RHBDD2 was determined by quantitative real time PCR in xenograft tumor tissues. **p < 0.01, ***p < 0.001, compared with NC mimics; ##*p* < 0.01, ###*p* < 0.001, compared with miR-4739 mimics

To investigate the impact of the miR-4739/RHBDD2 axis on DDP resistance in CC in vivo, a xenograft tumor model was established in nude mice. HeLa/DDP cells were transfected with either miR-4739 mimics alone or miR-4739 mimics along with RHBDD2 and then injected subcutaneously into BALB/c nude mice. After 2 weeks, when the tumor volume exceeded 100 mm^3^, the mice were treated with daily intraperitoneal injections of DDP at a dose of 20 mg/kg for 4 weeks. The results revealed that the miR-4739 mimics group exhibited slower tumor growth rates compared with the NC mimics group between day 28 and day 42. Notably, the overexpression of RHBDD2 reversed the effects of miR-4739 mimics (Fig. 6A). On day 42, the tumor size (Fig. 6B) and tumor weight (Fig. 6C) of HeLa/DDP cells overexpressing miR-4739 were significantly reduced after DDP treatment compared with HeLa/DDP cells without miR-4739 overexpression. Importantly, the effect of miR-4739 overexpression was counteracted when RHBDD2 was administered. Immunohistochemistry analysis demonstrated a decline in RHBDD2 expression in the miR-4739 mimics group, while an increase was observed in the group where miR-4739 mimics were combined with RHBDD2 in xenograft tumor tissues (Fig. 6D). Additionally, quantitative real-time PCR confirmed the upregulation of miR-4739 and downregulation of RHBDD2 in xenograft tumor tissues derived from the miR-4739 mimics group. Moreover, the overexpression of RHBDD2 reversed the downregulation of RHBDD2 (Fig. 6E). Collectively, these findings from the animal model align with the results obtained in vitro, underscoring the importance of the miR-4739/RHBDD2 axis in DDP resistance in CC.

## Discussion

Resistance to current chemotherapy drugs presents a significant hurdle in the effective treatment of cancer. DDP, an extensively used and highly potent chemotherapeutic agent, plays a crucial role in CC treatment. However, the frequent development of DDP resistance often leads to treatment failures [23]. Notably, recent evidence underscores notable distinctions in miRNA expression patterns between DDP-resistant cancer cells and their parental counterparts [24, 25]. In our study, we made the intriguing observation of down-regulated miR-4739 in DDP-resistant cancer cells lines and tumor tissues, which correlated with enhanced chemosensitivity. This finding aligns with prior research, reinforcing the notion of miR-4739 as a tumor suppressor. For instance, in pancreatic ductal adenocarcinoma (PDA), increased expression of miR-4739 has been shown to effectively inhibit cell growth and development in vitro [26]. Additionally, in gastric cancer, miR-4739 has been found to play a negative role in TYMSOS, thereby suppressing cell proliferation, migration, and invasion [27]. Furthermore, in the case of ESCC, the overexpression of miR-4739 has shown remarkable potential in inhibiting cell proliferation, migration, and invasion, while concurrently promoting apoptosis [17]. Collectively, these findings provide further evidence of the tumor-suppressive role of miR-4739 across multiple cancer types.

Continuing our investigation, a gain-of-function assay was performed to shed light on the impact of miR-4739 overexpression on chemosensitivity. The findings revealed a notable enhancement in the sensitivity of HeLa/DDP and SiHa/DDP cells to DDP upon the introduction of miR-4739. These results provide further support for the pivotal role of miR-4739 in sensitizing cells to DDP treatment. Furthermore, our study established RHBDD2 as a direct downstream target of miR-4739. Treatment with miR-4739 led to a significant reduction in RHBDD2 expression, and genetic silencing of RHBDD2 similarly resulted in improved chemosensitivity to DDP. These observations are consistent with previous studies demonstrating that the knockout of RHBDD2 reduces the apoptosis ratio in cancer cells, indicating the potential of RHBDD2 as a target for conventional chemotherapies [28]. Moreover, overexpression of RHBDD2 has been associated with the promotion of a chemo-resistant and invasive phenotype in rectal cancer tumors, achieved through the modulation of the unfolded protein response (UPR) and focal adhesion genes [22]. These collective findings underscore the significance of RHBDD2 as a prospective therapeutic target, emphasizing its role in facilitating chemoresistance and invasiveness in cancer. Consequently, considering our study findings along with those of other independent research groups, miR-4739 emerges as a critical modulator in the regulation of chemoresistance, highlighting its potential as a target for therapeutic intervention. While the involvement of other miR-4739 targets in DDP chemoresistance requires further investigation, our findings provide evidence that elevated RHBDD2 expression confers protection to CC cells against DDP treatment.

Crucially, our rescue experiments provided compelling evidence that the upregulation of RHBDD2 counteracted the regulatory effects of miR-4739 on DDP resistance in CC cells, both in vitro and in vivo. Notably, the overexpression of RHBDD2 reversed the downregulation of drug-resistance-related proteins (P-gp and MRP1) induced by miR-4739 mimics at the molecular level. P-gp (ABCB1) and MRP1 (ABCC1) are prominent ATP-binding cassette (ABC) transport proteins known to confer resistance to a wide range of anticancer agents, leading to multidrug resistance [29]. Previous studies have consistently reported the correlation between high levels of MDR1 and P-gp with chemotherapy resistance, lymph node metastasis, and reduced survival in patients with cancer [30]. In alignment with our research framework, a study by Li et al. [31] revealed that in DDP-resistant Eca-109 and TE1 cells, the expression levels of MRP1, ABCG1, ABCA1, and NLRP3 were all decreased by miR-495 mimics. This decrease was significantly reversed by overexpressing ATP7A. These collective findings further strengthen the understanding that dysregulation of ABC transporters plays a pivotal role in multidrug resistance, highlighting the intricate involvement of miRNAs in modulating the expression of these transporters.

## Conclusions

Overall, our study provides valuable insights into the molecular mechanisms underlying chemoresistance in CC and identifies the miR-4739/RHBDD2 axis as a potential therapeutic target. By targeting this axis, it may be possible to mitigate chemoresistance and enhance the efficacy of CC treatment. These findings have the potential to contribute to the development of novel therapeutic strategies for CC and the reduction of chemoresistance.

### Supplementary Information

**Additional file 1: Figure S1.** The effects of DDP treatment on the regulatory of miR-4739/RHBDD2 axis on DDP resistance in CC cells. The effects of DDP treatment on the regulatory of miR-4739 overexpression on colony formation (**A**), apoptosis (**B**), and migration (**C**) in HeLa/DDP and SiHa/DDP cells. The effects of DDP treatment on the regulatory of RHBDD2 knockdown on apoptosis (**A**), and migration (**B**) in HeLa/DDP and SiHa/DDP cells. **p < 0.01, ***p < 0.001, compared with DDP.

## Data Availability

The analyzed data sets generated during the present study are available from the corresponding author on reasonable request.

## Abbreviations

CC: Cervical cancer
DDP: Cisplatin
RHBDD2: Rhomboid domain containing 2
ATCC: American Type Culture Collection
DMEM: Dulbecco’s modified Eagle’s medium
FBS: Fetal bovine serum
3′-UTRs: 3′-untranslated regions
siRNAs: Small interfering RNAs
RIPA: Radioimmunoprecipitation assay
PVDF: Polyvinylidene fluoride
CCK-8: Cell Counting Kit-8
PI: Propidium iodide
ECL: Enhanced chemiluminescence
