# Supplementary figures and images for: Role of microRNA-4739 in enhancing cisplatin chemosensitivity by negative regulation of RHBDD2 in human cervical cancer cells
Source: Cell Mol Biol Lett. 2024 Jan 25;29:20. doi: 10.1186/s11658-024-00532-6 (PMC10809578; doi:10.1186/s11658-024-00532-6)

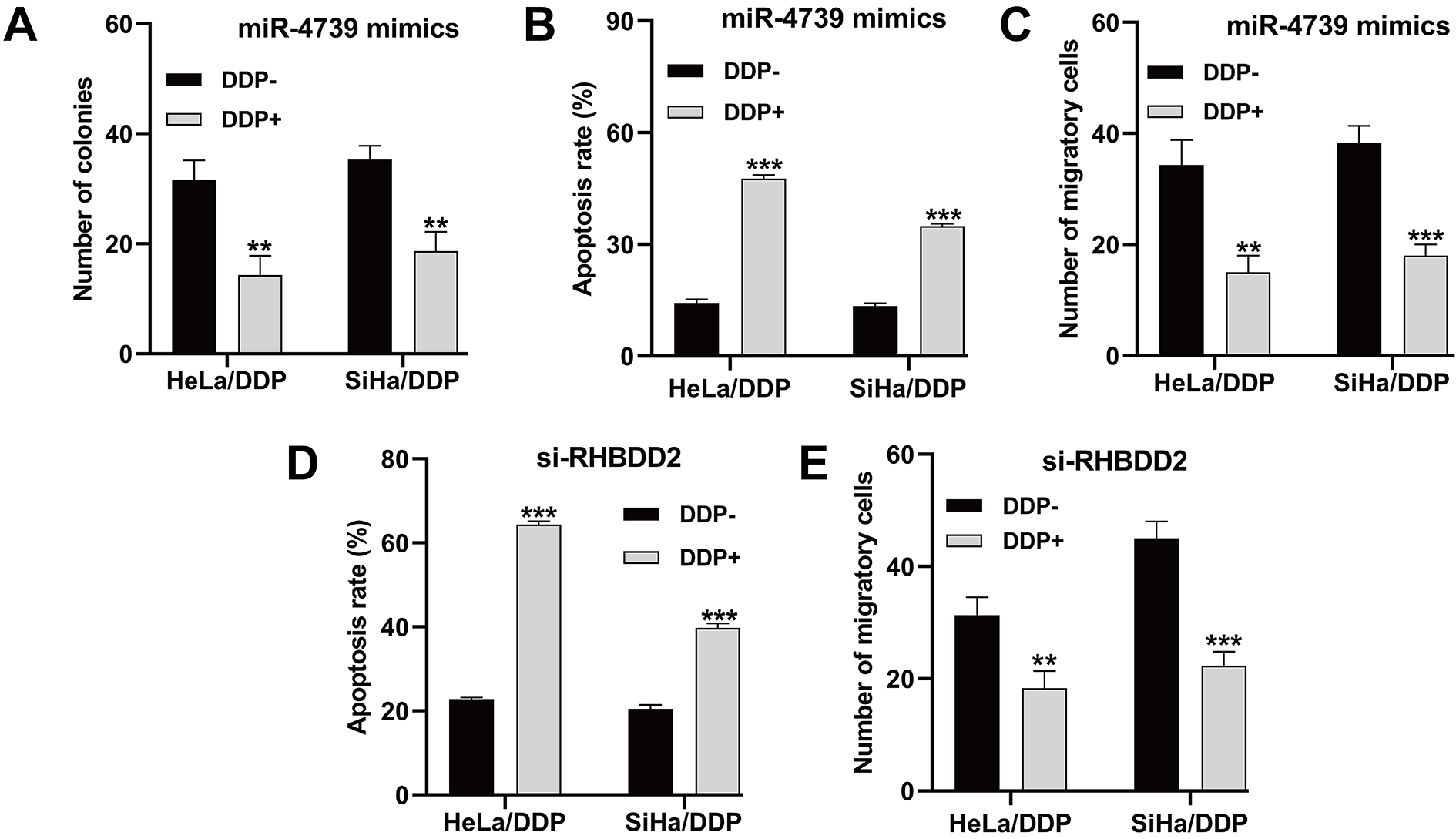

Supplement: Supplementary file 1 — Additional file 1: Figure S1. The effects of DDP treatment on the regulatory of miR-4739/RHBDD2 axis on DDP resistance in CC cells. The effects of DDP treatment on the regulatory of miR-4739 overexpression on colony formation (A), apoptosis (B), and migration (C) in HeLa/DDP and SiHa/DDP cells. The effects of DDP treatment on the regulatory of RHBDD2 knockdown on apoptosis (A), and migration (B) in HeLa/DDP and SiHa/DDP cells. **p < 0.01, ***p < 0.001, compared with DDP. [file 11658_2024_532_MOESM1_ESM.tif]
